# Comparison of cerebrospinal fluid space between probable normal pressure hydrocephalus and Alzheimer’s disease

**DOI:** 10.3389/fnagi.2023.1241237

**Published:** 2023-08-24

**Authors:** Hongliang Li, Chunyan Liu, Hong Tai, Youping Wei, Taizhong Shen, Qiong Yang, Keyang Zheng, Yan Xing

**Affiliations:** ^1^Department of Neurology, Aviation General Hospital, Beijing, China; ^2^Department of Medical Imaging, Aviation General Hospital, Beijing, China; ^3^Department of Rehabilitation, Aviation General Hospital, Beijing, China; ^4^Department of Cardiovascular Medicine, Capital Medical University Affiliated Anzhen Hospital, Beijing, China

**Keywords:** idiopathic normal pressure hydrocephalus, voxel-based morphometry, Alzheimer’s disease, cerebrospinal fluid, differential diagnosis

## Abstract

**Introduction:**

Idiopathic normal pressure hydrocephalus (INPH) is a potentially reversible syndrome characterized by complex symptoms, difficulty in diagnosis and a lack of detailed clinical description, and it is difficult to distinguish from Alzheimer’s disease (AD). The objective of this study was to design a method for measuring the actual amount of hydrocephalus in patients with INPH and to evaluate INPH.

**Methods:**

All subjects underwent a 3D T1-weighted MRI. Statistical parametric mapping 12 was used for preprocessing images, statistical analysis, and voxel-based morphometric gray matter (GM) volume, white matter (WM) volume, and cerebrospinal fluid (CSF) volume analysis. The demographic and clinical characteristics of the groups were compared using a *t*-test for continuous variables and a chi-square test for categorical variables. Pearson’s correlation analysis and Bonferroni’s statistic-corrected one-way ANOVA were used to determine the relationship among demographic variables. Receiver operating characteristic (ROC) curves were used to assess the accuracy of the callosal angle (CA), WM ratio, and CSF ratio in distinguishing probable INPH from AD.

**Results:**

The study included 42 patients with INPH, 32 patients with AD, and 24 healthy control subjects (HCs). There were no differences among the three groups in basic characteristics except for Mini-Mental State Examination (MMSE). There was a correlation between the intracranial CSF ratio and CA. The WM ratio and CSF ratio in patients with INPH and AD were statistically different. Furthermore, the combination of CA, WM ratio, and CSF ratio had a greater differential diagnostic value between INPH and AD patients than CA alone.

**Conclusion:**

INPH can be accurately assessed by measuring intracranial CSF ratio, and the addition of WM ratio and CSF ratio significantly improved the differential diagnostic value of probable INPH from AD compared to CA alone.

## 1. Introduction

Idiopathic normal pressure hydrocephalus (INPH) is a potentially reversible syndrome characterized clinically by ventricular enlargement (enlarged ventricles), cognitive impairment, gait disorders, and urinary incontinence (Williams and Relkin, 2013; Chunyan et al., 2021; Nakajima et al., 2021). Alzheimer’s disease (AD) shares common features with INPH in clinical appearances, laboratories, and imaging, such as executive dysfunction, impairment in attention and short-term memory in cognition (Chunyan et al., 2021). INPH and AD are both likely to happen in the elderly population but INPH is a reversible neurological disease (Yamada et al., 2016). Therefore, accurate diagnosis of INPH and differentiation between INPH and AD with brain atrophy hold significant importance for appropriate treatment strategies.

Imaging techniques can reveal specific characteristics such as gray matter cortical atrophy, white matter degeneration, and ventriculomegaly, which are indicative of both INPH and AD, albeit with varying degrees. One particular imaging parameter of interest is the callosal angle (CA), defined as the angle between the superior walls of the ventricles formed by the left and right parts of the corpus callosum. Previous research has shown that CA angles less than 90 degrees not only serve as a diagnostic marker for INPH but also predict surgical outcomes (Virhammar et al., 2014a). However, CA measurements rely on visual interpretation, making them susceptible to subjective errors by technicians and evaluators (Benedetto et al., 2017; Han et al., 2022). Therefore, there is a need for automated volumetric measurements to provide a more objective and reliable assessment of regional brain volume (van den Heuvel et al., 2006).

In recent years, a novel method called ITK-SNAP has been developed for measuring ventricular volume (Lindberg et al., 2018; Neikter et al., 2020). However, this approach is time-consuming, requiring at least 8 h for calculations. In contrast, the voxel-based morphometry (VBM) Toolbox SPM12 offers a faster alternative, taking only a few minutes to perform the same measurements. With the VBM method, brain tissue scans are automatically segmented into gray matter (GM), white matter (WM), and cerebrospinal fluid (CSF). The volumes of GM, WM, and CSF are then calculated separately and combined to obtain the total intracranial volume (TIV) (Manniche et al., 2019; Mao et al., 2020). This is achieved by utilizing a prior probability map or “Bayesian prior,” which represents the spatial distribution of different tissue types in healthy individuals. A mixed-model clustering analysis is applied to determine the voxel intensity distribution of specific tissue types (Yushkevich et al., 2006; Akudjedu et al., 2018). In our study, we aimed to assess whether the GM ratio (GM volume/TIV), WM ratio (WM volume/TIV), and CSF ratio (CSF volume/TIV) could be utilized for diagnosing probable INPH and differentiating between probable INPH and AD.

## 2. Materials and methods

### 2.1. Participants

From May 2018 to November 2021, a total of 67 patients with possible INPH and 32 patients with AD were recruited from the inpatient clinic of the Department of Neurology at the Aviation General Hospital. Additionally, 24 healthy control subjects (HCs) were recruited from local health screening centers. All participants provided written informed consent, and the study was approved by the Ethics Committee of the Aviation General Hospital (HK2018-03-20).

**FIGURE 1:**
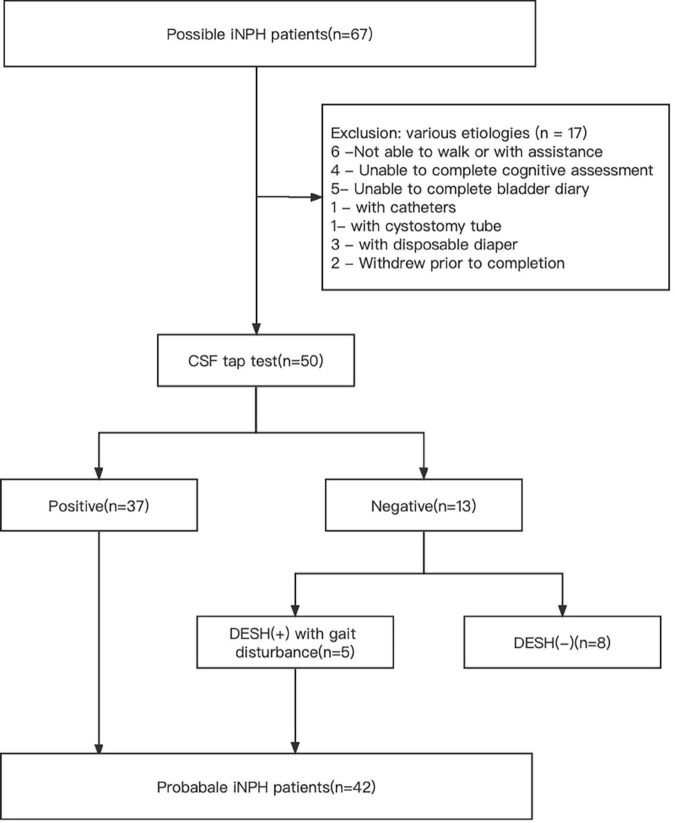
Flowchart for enrolling probable INPH patients in this study.

For the analysis of this study, probable INPH patients were subjected to exclusion criteria, which included (a) secondary hydrocephalus and (b) inability to complete assessments related to gait disturbance, cognitive impairment, and urinary incontinence. Among the 67 patients initially considered, 50 met the inclusion criteria, while the remaining 17 were excluded from the study. These 50 patients underwent cerebrospinal fluid tap test (CSF TT). Positive responses to CSF TT were determined based on the following criteria: a 10% or greater improvement in time on the 3-mTUG test, a 20% improvement in steps or time on the 10-MWT, a 10% improvement in both, or an increase of 3 or more points on the Mini-Mental State Examination (MMSE) scale. After CSF TT, 37 patients demonstrated a positive response and were diagnosed with probable INPH. Additionally, among the 13 patients who showed a negative response, 5 patients with gait disturbance and disproportionately enlarged subarachnoid space hydrocephalus (DESH) were diagnosed with probable INPH. Ultimately, a total of 42 patients with probable INPH were included in the study (Figure 1). The healthy control subjects had no history of neurological or psychiatric diseases, cerebrovascular disease, head trauma, substance abuse, or the use of medications that could impact the central nervous system.

### 2.2. Assessment of probable INPH and Alzheimer’s disease

The diagnosis of probable INPH in this study was based on the third edition of the Japanese INPH guidelines (Nakajima et al., 2021), and the inclusion criteria were as follows. (a) Presence of more than one symptom in the clinical trial. Gait disturbance, cognitive impairment, and urinary incontinence, (b) the above clinical symptoms could not be fully explained by other neurological or non-neurological diseases, (c) the preceding disease that could lead to ventricular dilatation (including subarachnoid hemorrhage, meningitis head injury, congenital/developmental hydrocephalus, and aqueductal stenosis) were not evident, (d) CSF pressure was 200 mmH^2O or lower and CSF levels are normal, (e) neuroimaging features of hyperconvex/midline surface sulcus and narrowing of the subarachnoid space with gait disturbance. Additional gait-related features included smaller gait, shuffling, instability during walking, increased instability during turning, or improvement in symptoms after CSF TT (Nakajima et al., 2021).

On the other hand, the diagnosis of AD was made according to the criteria established by the National Institute of Neurological and Communicative Disorders and Stroke-Alzheimer’s Disease and Related Disorders Association (NINCDS-ADRDA) (McKhann et al., 1984; Dubois et al., 2014).

### 2.3. Cerebrospinal fluid tap tests

The day prior to the CSF TT, all patients underwent evaluation for three main characteristics, referred to as the Pre-CSF TT assessment. Subsequently, the CSF TT was performed by removing 30 ml of CSF or until no more CSF could be extracted (Marmarou et al., 2005). Following the CSF TT, assessments were conducted to evaluate changes in gait, cognition, and bladder function at specific time intervals. Gait changes were assessed at 4 h, 1 day, 2 days, and 3 days post-CSF TT. Cognitive changes were evaluated 1 week after the CSF TT, while bladder function was assessed 2 days after the procedure. These assessments were carried out by two experienced assessors who were blinded to the patients’ clinical diagnoses. During the gait assessment, patients were recorded while walking. The MMSE and the Montreal Cognitive Assessment (MoCA) (Folstein et al., 1975) were used for cognitive evaluation, while a bladder diary was employed to assess bladder function. Instead of using a fixed time point for all patients, the post-CSF TT index was determined as the highest value of individual gait improvement observed after the CSF TT.

### 2.4. Image acquisition and preprocessing

All participants underwent structural three-dimensional T1-weighted imaging (3D-T1WI) in the sagittal plane using a 3.0 Tesla scanner (Discovery MR750, GE Healthcare, UK). The imaging protocol consisted of a whole-brain 3D-T1WI fast spoiled gradient echo sequence with the following parameters: repetition time 8 ms, echo time 3 ms, flip angle 15°, sagittal slice 178, field of view 256 mm, and voxel size 1 × 1 × 1 mm. The scans were performed using various scanners, including Discovery, SignaHDxt, and Achieva.

The raw imaging data were anonymized and transferred from the scanner in DICOM format. Subsequently, the data were converted to nii format, and the reoriented coordinate origin was manually corrected using MATLAB 2014.b (MathWorks, Natick, MA) in the Windows 10 operating system. The correction was based on the anterior commissure - posterior commissure line in Statistical Parametric Mapping (SPM12, Functional Imaging Laboratory, University College London, London, UK). Prior to segmentation, a spatial normalization technique was applied to improve the accuracy of brain tissue segmentation (Ashburner and Friston, 2000; Lancaster et al., 2000).

### 2.5. Image analysis

CA measurements were performed by experienced radiologists for each subject based on T1 MRI images. CA refers to the angle formed by the corpus callosum between the lateral ventricles in a coronal plane that is perpendicular to the anterior-posterior commissure (Mori et al., 2012; Miskin et al., 2017).

Three-dimensional T1-weighted images were processed using VBM Toolbox SPM12 to process (Ashburner and Friston, 2000). This processing involved automatic segmentation of the brain tissue scans into GM, WM, and CSF. The total volumes of GM, WM, and CSF were then calculated individually and combined to obtain the TIV. Additionally, the ratios of GM, WM, and CSF to TIV were calculated to assess their respective contributions.

### 2.6. Statistical analysis

The normality of the variables was assessed using the Kolmogorov-Smirnov test. A *t*-test was conducted to compare continuous variables, while a chi-square test was used for categorical variables to compare the demographic and clinical characteristics between groups. Pearson’s correlation analysis and Bonferroni’s statistic corrected one-way ANOVA were employed to examine relationships between demographic variables.

To evaluate the diagnostic accuracy of CA, WM ratio, and CSF ratio in distinguishing probable INPH from AD, receiver operating characteristic (ROC) curves were constructed. Area under the curve (AUC), sensitivity, specificity, and cutoff levels were calculated from the ROC curves. The Delong test was employed to compare the differences among these ROC curves.

All statistical analyses were performed using the R statistical software package (The R Foundation).^1^ The significance level was set at *P* < 0.05, and data were presented as mean ± standard deviation. A *p*-value less than 0.05 was considered statistically significant.

## 3. Results

### 3.1. Baseline characteristics of the study population

A total of 98 participants were included in the study, with a mean age of 74.4 ± 7.7 years. Out of these patients, 50 (51.02%) were male. The patients were divided into three groups based on their respective diseases, and Table 1 provides an overview of the characteristics of the patients.

**TABLE 1:** Clinical characteristics of the study population.

	AD	HCs	Probable INPH	*p*-value
*N*	32	24	42	
Male, *n* (%)	13 (40.6%)	13 (54.2%)	24 (57.1%)	0.348
Age (year), mean ± SD	76.8 ± 8.6	75.9 ± 6.3	74.8 ± 7.3	0.534
MMSE	20.0 ± 4.5	29.2 ± 1.0	19.3 ± 7.5	<0.001
Hypertension, *n* (%)	16 (50.0%)	11 (45.8%)	28 (66.7%)	0.181
Coronary disease, *n* (%)	8 (25.0%)	4 (16.7%)	10 (23.8%)	0.732
Hyperlipidemia, *n* (%)	13 (40.6%)	9 (37.5%)	19 (45.2%)	0.817
Diabetes mellitus, *n* (%)	8 (25.0%)	6 (25.0%)	14 (33.3%)	0.665
Carotid arteriosclerosis, *n* (%)	11 (34.4%)	6 (25.0%)	14 (33.3%)	0.720
Smoking, *n* (%)	5 (15.6%)	5 (20.8%)	9 (21.4%)	0.805
Alcohol, *n* (%)	4 (12.5%)	2 (8.3%)	5 (11.9%)	0.872

MMSE, Mini Mental State Examination; AD, Alzheimer’s disease; HCs, healthy control subjects; INPH, idiopathic normal pressure hydrocephalus.

There was no significant difference observed in the average age and sex ratio among the three groups (*p* = 0.348 for male and *p* = 0.534 for age). However, the HCs group had a higher MMSE score compared to both the AD and INPH patients (*p* < 0.001). Additionally, there were no significant differences found in other variables, including basic disease history, smoking, and alcohol consumption, among the three groups (all *p* > 0.05).

### 3.2. Neuroimaging assessment and volumetric comparison

**FIGURE 2:**
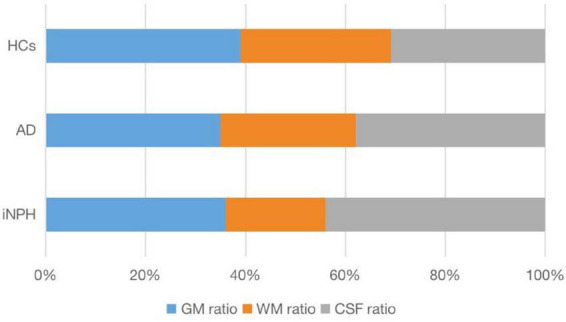
The average ratio of GM, WM, and CSF in patients with probable INPH, AD, and HCs.

Table 2 presents the mean values and standard deviations (SDs) for the measurements of neuroimaging indicators and Figure 2 illustrates the average ratios of GM, WM, and CSF in patients with probable INPH, AD, and HCs.

**TABLE 2:** Mean values and SDs for measurements of neuroimaging indicators.

Group	Probable INPH	AD	HCs	*p*-value^a^	*p*-value^b^
CA (degree)	99.32 ± 18.41	119.28 ± 10.53	122.12 ± 5.91	<0.001	<0.001
GM volume	0.57 ± 0.08	0.51 ± 0.06	0.55 ± 0.05	0.001	0.483
WM volume	0.32 ± 0.12	0.39 ± 0.05	0.43 ± 0.05	0.002	<0.001
CSF volume	0.69 ± 0.11	0.55 ± 0.08	0.45 ± 0.06	<0.001	<0.001
TIV volume	1.58 ± 0.17	1.45 ± 0.13	1.43 ± 0.12	<0.001	<0.001
GM ratio	0.36 ± 0.05	0.35 ± 0.03	0.39 ± 0.02	0.361	0.03
WM ratio	0.20 ± 0.07	0.27 ± 0.03	0.30 ± 0.02	<0.001	<0.001
CSF ratio	0.44 ± 0.05	0.38 ± 0.04	0.31 ± 0.03	<0.001	<0.001

AD, Alzheimer’s Disease; HCs, healthy control subjects; INPH, idiopathic normal pressure hydrocephalus; CA, callosal angle; GM, gray matter; WM, white matter; CSF, cerebrospinal fluid.

^a^*p*-value: indicates the probability value of INPH vs AD.

^b^*p*-value: indicates the probability value of INPH vs HC.

The CA values for the probable INPH, AD, and HC groups were 99.32 ± 18.41, 119.28 ± 10.53, and 122.12 ± 5.91, respectively. The probable INPH patients had significantly smaller CA values compared to the AD and HC groups (*p* < 0.001 for both comparisons). In terms of other neuroimaging indicators, the probable INPH patient group showed significantly lower WM volume and higher CSF volume and TIV volume compared to both the HC and AD groups (all *p* < 0.05). However, when considering GM volume, probable INPH patients had significantly higher levels compared to AD patients (*p* = 0.001), while there was no significant difference between probable INPH patients and HCs (*p* = 0.483).

After adjusting for TIV as a covariate to account for individual differences, the differences between the three patient groups remained consistent. The CSF ratio for the probable INPH, AD, and HC groups were 0.44 ± 0.05, 0.38 ± 0.04, and 0.31 ± 0.03, respectively. Due to significant CSF accumulation in probable INPH, these patients had a higher CSF ratio compared to both the HC and AD groups (*p* < 0.001). However, the CSF ratio was also elevated in AD patients due to brain atrophy (*p* < 0.001 compared to HCs). Probable INPH patients had a higher WM ratio compared to both AD patients and HCs (*p* < 0.001). Although there was a difference between probable INPH patients and HCs (*p* = 0.03), no significant difference was observed between patients with probable INPH and AD (*p* = 0.361) in terms of WM ratio. The summarized results are presented in Figure 2 and Table 1.

### 3.3. The correlation between CSF ratio and CA

**FIGURE 3:**
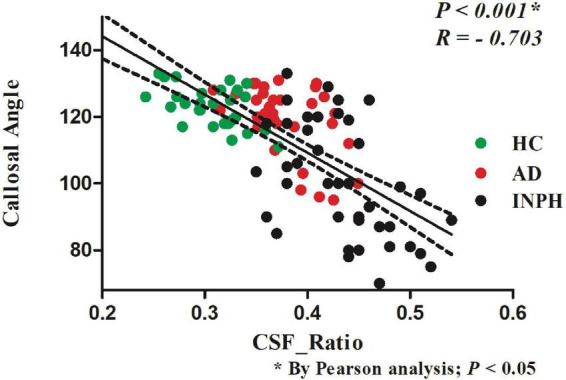
The relationship between the CSF volume ratio and the callosal angle index of the patients in the indicated groups.

Figure 3 illustrates the negative correlation between the CSF ratio and CA in all three groups (*R* = −0.703, *p* < 0.001, Pearson’s correlation). The CSF ratio in probable INPH patients (0.44 ± 0.05) was significantly higher than that in both the AD group (0.38 ± 0.04) and the HCs group (0.31 ± 0.03) (*p* < 0.001, Pearson’s correlation).

### 3.4. The differential diagnosis value of probable INPH from AD among different indicators by receiver operating characteristic graph curves

**FIGURE 4:**
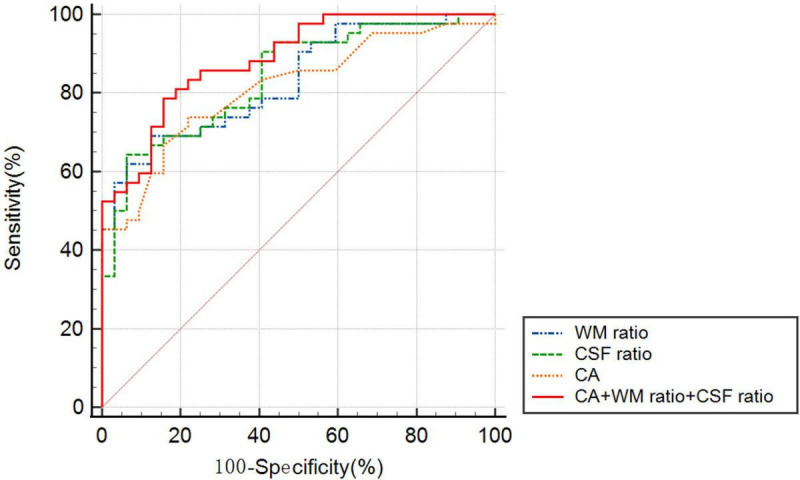
The receiver operating characteristic graph curves of callosal angle, WM ratio, CSF ratio and combined indicator for the differential diagnosis of probable INPH from AD.

Figure 4 presents the ROC curves for CA, WM ratio, CSF ratio, and their combination. Table 3 provides the corresponding diagnostic performance metrics. CA demonstrated relatively higher sensitivity (0.7381), while CSF ratio exhibited relatively higher specificity (0.9375). All three indicators, namely WM ratio (AUC = 0.8095, 95%CI 0.7128 to 0.9063), CSF ratio (AUC = 0.8393, 95%CI 0.7503 to 0.9283), and CA (AUC = 0.8121, 95%CI 0.7153 to 0.9089), exhibited substantial potential for the differential diagnosis of probable INPH from AD. Moreover, there were no statistically significant differences observed among these three indicators (*p* = 0.7385 for CA vs. WM ratio and *p* = 0.6236 for CA vs. CSF ratio). These findings indicate that CSF ratio and WM ratio have comparable diagnostic values in distinguishing probable INPH from AD.

**TABLE 3:** Predictive value of callosal angle, WM ratio, CSF ratio and combined indicator for the differential diagnosis of probable INPH from AD.

Group	AUC	95%Cl	Cut-off	Sensitivity	Specificity	PPV	NPV
CA	0.8121	0.7153 to 0.9089	116.5000	0.7381	0.7812	0.8158	0.6944
WM ratio	0.8311^a^	0.7128 to 0.9063	0.2430	0.6667	0.8750	0.875	0.6667
CSF ratio	0.8393^b^	0.7503 to 0.9283	0.4279	0.6429	0.9375	0.931	0.6667
WM ratio + CSF ratio + CA	0.8869^c^	0.8153 to 0.9585	NA	0.7857	0.8438	0.8684	0.7500

Combined Indicator: combine callosal angle, WM ratio and CSF ratio.

^a^*p* = 0.7385 vs CA;

^b^*p* = 0.6236 vs CA;

^c^*p* = 0.0461 vs CA.

Moreover, when combining all three indicators (CA, WM ratio, and CSF ratio), the resulting AUC was statistically higher than that of CA alone (*p* = 0.0461). This suggests that the combination of CA, WM ratio, and CSF ratio provides an improved diagnostic value compared to using CA alone.

## 4. Discussion

In this study, we investigated the diagnostic performance and clinical significance of automated volumetric measurement of intracranial CSF for diagnosing probable INPH, and compared it with traditional neuroimaging markers. Our findings revealed a strong correlation between intracranial CSF ratio and the conventional image biomarker of CA. Moreover, we identified WM ratio and CSF ratio as two indicators that can effectively differentiate between INPH and AD. Importantly, the inclusion of WM ratio and CSF ratio significantly enhanced the diagnostic value for distinguishing probable INPH from AD, beyond the use of CA alone. These results highlighted the potential clinical impact of whole-brain automated volumetric measurements in the diagnosis and differentiation of INPH and AD.

Timely detection and intervention of probable INPH are crucial, as it represents a potentially treatable cause of dementia (Wu et al., 2019). However, the clinical diagnosis of probable INPH poses challenges for both neurologists and radiologists. INPH is clinically characterized by a triad of gait ataxia, urinary incontinence, and dementia. It is important to note that several untreatable disorders exhibit the same clinical trial, making diagnosis difficult. For instance, Parkinson’s disease (PD), Dementia with Lewy bodies (DLB), Progressive supranuclear palsy (PSP), and Multiple system atrophy (MSA) present similar symptoms (Gallia et al., 2006; Williams and Malm, 2016). Furthermore, a multitude of other disorders imitate one or two of the three key clinical diagnostic criteria associated with probable INPH, including other forms of dementia such as AD and frontotemporal dementia (FTD) (Gallia et al., 2006; Nassar and Lippa, 2016; Pomeraniec et al., 2016; Williams and Malm, 2016). This complexity further complicates accurate disease diagnosis.

INPH and AD exhibit similar clinical presentations, characterized by neurodegeneration, cognitive decline, physical deterioration, and sleep disturbances (Rauchs et al., 2008; Roman et al., 2019). Consequently, AD has emerged as the primary differential diagnosis for INPH (Laitera et al., 2015). Apart from clinical symptoms, distinguishing between INPH and AD relies on morphological assessment through CT or MRI (Wu et al., 2019; Nakajima et al., 2021). The differentiation of INPH from AD and other neurodegenerative diseases can sometimes be determined by the presence of ventriculomegaly, significantly reduced CA, and notably increased Evans’ index (EI) (Hoshi et al., 2012; Hiraoka et al., 2015; Yokota et al., 2019). The diagnostic value of CA has been demonstrated in multiple studies (Virhammar et al., 2014a,b). CA can differentiate between INPH and AD with a sensitivity of 97%, specificity of 88%, and positive predictive value of 93% at a cutoff value of 90° (Ishii et al., 2008). However, it is important to note that CA measurements can be significantly affected by the position and method of measurement, emphasizing the need for standardization of the measurement protocol (Nakajima et al., 2021).

Radiological differentiation is often challenging due to the overlapping symptoms of multiple neurodegenerative diseases. T1-weighted brain MRI can distinguish between INPH and non-INPH AD patients or elderly HC volunteers with an accuracy of 68–78%, but the agreement is only fair (intraclass correlation coefficient [ICC], 0.51; 95% confidence interval: 0.34, 0.66) (Lee et al., 2010). However, the evaluation of structural images is susceptible to subjective errors due to individual differences among imaging technicians. Moreover, there are significant individual differences in INPH itself. For instance, while some patients may have enlarged ventricles, their frontal angle may not be significantly expanded, whereas the occipital angle may show notable expansion. Therefore, the calculated EI may not accurately reflect the actual ventricular expansion in such patients. Additionally, ventricular enlargement can also be observed in AD as a consequence of severe cerebral atrophy, further complicating the radiographic identification of INPH (Tarasoff-Conway et al., 2015).

The semi-automatic method of objectively segmenting intracranial components allows for the precise measurement of CSF content, offering greater accuracy in evaluating hydrocephalus compared to conventional imaging parameters such as EI and CA (van den Heuvel et al., 2006). Yamashita, as early as 2009, employed automatic segmentation to measure CSF content in patients with hydrocephalus (Yamashita et al., 2010). While ITK-SNAP was developed as a new method for measuring ventricular volumes, its complex, expensive, and time-consuming procedure renders it impractical for large-scale epidemiological and clinical studies (Williams and Relkin, 2013; Lindberg et al., 2018; Neikter et al., 2020). In contrast, spatially normalized three-dimensional (3D) T1-weighted MR images provide a more convenient and accessible alternative, which was utilized in the present study to collect data on GM volume, WM volume, and CSF volume. Neikter et al. (2020) confirmed the correlation between brain volume and EI by employing pure CSF instead of whole brain volume to account for individual differences. Similarly, we observed a strong correlation between the CSF ratio and CA. Furthermore, the findings of this study demonstrated that the combination of CA, WM ratio, and CSF ratio exhibited superior diagnostic value compared to CA alone in distinguishing between INPH and AD, potentially enhancing the accuracy of clinical diagnosis for probable INPH.

There are several limitations that should be acknowledged in this study. Firstly, the gold standard for diagnosing INPH is positive shunt surgery. However, since only a small number of INPH patients included in this study underwent shunt surgery, it was not utilized as the diagnostic standard in this paper. Secondly, while CSF volume measurement can accurately assess intracranial hydrocephalus, the potential impact of microenvironmental deterioration (e.g., periventricular white matter edema) remains unknown, and further verification is required to establish the effectiveness of this method. Lastly, the sample size in this study was small, which calls for caution in the application of CSF volume for hydrocephalus evaluation.

## 5. Conclusion

In this study, we employed the VBM Toolbox SPM12 to process three-dimensional T1-weighted images. Through this method, we discovered a correlation between the intracranial CSF ratio and the traditional image biomarkers CA. By measuring the WM ratio and CSF ratio, INPH could be accurately evaluated. Moreover, the diagnostic value of CA could be improved by incorporating the WM ratio and CSF ratio. This approach was straightforward and user-friendly, saving significant time and being more suitable for clinical implementation. However, further studies are required to develop precise and comprehensive evaluation methods for INPH.

## Data availability statement

The raw data supporting the conclusions of this article will be made available by the authors, without undue reservation.

## Ethics statement

The studies involving humans were approved by the Ethical Committee of the Aviation General Hospital (HK2018-03-20). The studies were conducted in accordance with the local legislation and institutional requirements. Written informed consent for participation was not required from the participants or the participants’ legal guardians/next of kin in accordance with the national legislation and institutional requirements.

## Author contributions

YX and HL organized the research project. CL and QY oversaw the subjects’ recruitment and execution. HT and KZ executed the research project and statistical analysis and wrote the first draft of the manuscript. YW and TS were responsible for scanning the subjects and supporting the image technology. HT was responsible for the subjects’ characterization and sample collection. All authors contributed and approved the final manuscript.

## Footnotes

1
http://www.R-project.org
