# Corrigendum: Comparison of cerebrospinal fluid space between probable normal pressure hydrocephalus and Alzheimer's disease

**DOI:** 10.3389/fnagi.2024.1378918

**Published:** 2024-02-13

**Authors:** Hongliang Li, Chunyan Liu, Hong Tai, Youping Wei, Taizhong Shen, Qiong Yang, Keyang Zheng, Yan Xing

**Affiliations:** ^1^Department of Neurology, Aviation General Hospital, Beijing, China; ^2^Department of Medical Imaging, Aviation General Hospital, Beijing, China; ^3^Department of Rehabilitation, Aviation General Hospital, Beijing, China; ^4^Department of Cardiovascular Medicine, Capital Medical University Affiliated Anzhen Hospital, Beijing, China

**Keywords:** idiopathic normal pressure hydrocephalus, voxel-based morphometry, Alzheimer's disease, cerebrospinal fluid, differential diagnosis

In the published article, there was an error in the Funding statement. No Funding was displayed. The correct Funding statement appears below.

